# The Bacterial Mucosal Immunotherapy MV130 Protects Against SARS-CoV-2 Infection and Improves COVID-19 Vaccines Immunogenicity

**DOI:** 10.3389/fimmu.2021.748103

**Published:** 2021-11-18

**Authors:** Carlos del Fresno, Juan García-Arriaza, Sarai Martínez-Cano, Ignacio Heras-Murillo, Aitor Jarit-Cabanillas, Joaquín Amores-Iniesta, Paola Brandi, Gillian Dunphy, Carmen Suay-Corredera, Maria Rosaria Pricolo, Natalia Vicente, Andrés López-Perrote, Sofía Cabezudo, Ana González-Corpas, Oscar Llorca, Jorge Alegre-Cebollada, Urtzi Garaigorta, Pablo Gastaminza, Mariano Esteban, David Sancho

**Affiliations:** ^1^ Department of Myocardial Pathophysiology, Centro Nacional de Investigaciones Cardiovasculares (CNIC), Madrid, Spain; ^2^ Department of Infectious Diseases and Immunity, Instituto de Investigación Biomédica del Hospital Universitario la Paz (IdiPAZ), Madrid, Spain; ^3^ Department of Molecular and Cellular Biology, Centro Nacional de Biotecnología (CNB), Consejo Superior de Investigaciones Científicas (CSIC), Madrid, Spain; ^4^ R&D Department, Inmunotek S.L., Alcalá de Henares, Spain; ^5^ Structural Biology Department, Centro Nacional de Investigaciones Oncológicas (CNIO), Madrid, Spain

**Keywords:** innate immunity, viral infections, polybacterial mucosal immunotherapy, SARS-CoV-2, vaccine immunogenicity

## Abstract

COVID-19-specific vaccines are efficient prophylactic weapons against SARS-CoV-2 virus. However, boosting innate responses may represent an innovative way to immediately fight future emerging viral infections or boost vaccines. MV130 is a mucosal immunotherapy, based on a mixture of whole heat-inactivated bacteria, that has shown clinical efficacy against recurrent viral respiratory infections. Herein, we show that the prophylactic intranasal administration of this immunotherapy confers heterologous protection against SARS-CoV-2 infection in susceptible K18-hACE2 mice. Furthermore, in C57BL/6 mice, prophylactic administration of MV130 improves the immunogenicity of two different COVID-19 vaccine formulations targeting the SARS-CoV-2 spike (S) protein, inoculated either intramuscularly or intranasally. Independently of the vaccine candidate and vaccination route used, intranasal prophylaxis with MV130 boosted S-specific responses, including CD8^+^-T cell activation and the production of S-specific mucosal IgA antibodies. Therefore, the bacterial mucosal immunotherapy MV130 protects against SARS-CoV-2 infection and improves COVID-19 vaccines immunogenicity.

## 1 Introduction

Respiratory infection by coronavirus SARS-CoV-2 has caused the COVID-19 pandemic. Vaccines are being developed at an unprecedented speed as an effective prophylaxis. Vaccines based on messenger RNA (1, 2) or viral vectors (3, 4) expressing the SARS-CoV-2 spike (S) protein showed efficacies between 60 and 95%. Among them, we have developed a novel COVID-19 vaccine candidate based on the Modified Vaccinia Virus Ankara (MVA) poxvirus vector expressing the entire SARS-CoV-2 S protein (termed MVA-CoV2-S or MVA-S) (5). This vaccine candidate, induced robust SARS-CoV-2-specific T-cell and humoral immune responses and was fully effective against lethal SARS-CoV-2 infection when administered in one or two doses to SARS-CoV-2-sensitive K18-hACE2 mice, with one dose providing less robust protection (5).

The potential need for improving efficacy of some COVID-19 vaccines after just one dose or against new variants, and the potential of future outbreaks of newly emerging viruses present the need for alternative prophylactic approaches. Immune therapies that induce heterologous protection are under investigation, as their success does not strictly rely on the specific recognition of cognate antigens that may be unknown at the time of intervention. Such approaches target both innate and adaptive immune responses in order to bring them to a “trained” status (6).

Among the immune therapy strategies to generate heterologous protection, the mucosal formulation MV130, a mixture of whole heat-inactivated bacteria, has been shown to be clinically effective against wheezing attacks in children, a respiratory pathology mostly caused by viral infections (7). Interestingly, patients suffering recurrent respiratory infections that received MV130 showed enhanced lymphoproliferative responses against influenza antigens (8), supporting the notion that MV130 could boost antigen-specific responses. Herein, we show that prophylactic intranasal immunotherapy with MV130 confers heterologous protection against SARS-CoV-2 infection in susceptible K18-hACE2 mice and enhances antigen-specific responses triggered by diverse vaccination strategies against COVID-19.

## 2 Materials and Methods

### 2.1 Mouse Strains

Mice were bred at CNIC under specific pathogen-free conditions. Mouse strains include C57BL/6 mice and K18-hACE2 mice [B6.Cg-Tg(K18-ACE2)2Prlmn], both from the Jackson Laboratory. ﻿We used age-matched 7- to 9-week-old mice. Efficacy experiments were performed in the biosafety level 3 (BSL-3) facilities at the Centro de Investigación en Sanidad Animal (CISA)-Instituto Nacional De Investigaciones Agrarias (INIA) (Madrid, Spain). Experiments were approved by the animal ethics committees at CNIC and CISA and by the Division of Animal Protection of the Comunidad de Madrid (PROEX 169.4/20, and PROEX14/16). Animal procedures conformed to Spanish law under the Royal Decree (RD 53/2013) and performed in accordance with EU Directive 2010/63EU and Recommendation 2007/526/EC.

### 2.2 Excipient and MV130 Administration

**Figure 1:**
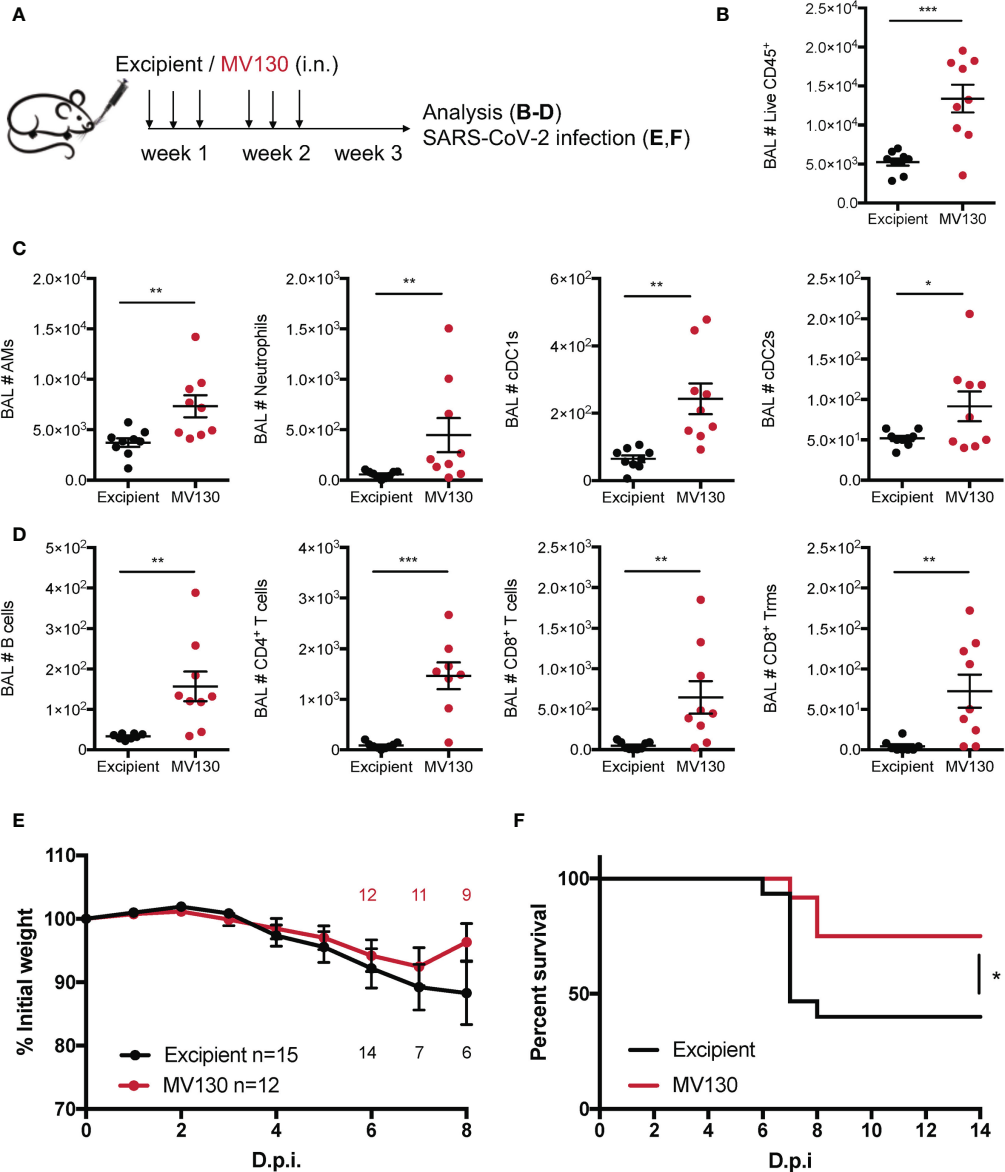
MV130 prophylactic administration protects against SARS-CoV-2 infection. **(A)** Scheme of immunotherapy administration. Mice were i.n. administered with excipient or MV130 three times per week for 2 weeks. After a resting week, mice were analyzed or infected. **(B–D)** Bronchoalveolar lavage (BAL) was obtained from excipient and MV130-treated C57BL/6 mice. Quantitative analysis of total live CD45^+^ cells **(B)**, different myeloid **(C)** or lymphoid **(D)** immune populations identified by FACS. AMs, alveolar macrophages; cDC1s, type 1 conventional dendritic cells; cDC2s, type 2 conventional dendritic cells. N=2. **(E, F)** K18-hACE2 mice received excipient or MV130 as indicated in **(A)**. One week later they were i.n. infected with 10^4^ PFU of SARS-CoV-2 (MAD6 strain). Weight **(E)** and survival **(F)** were monitored daily. **(E)** Numbers in the graph indicate remaining alive mice. N=2. (n=15 excipient; n=12 MV130). D.p.i., days post infection. **(B–E)** Data shown as mean ± SEM. *p < 0.05; **p< 0.01; ***p < 0.001, Student’s t test comparing excipient *versus* MV130. **(F)** *p < 0.05, Mantel-Cox test.

Mice were intranasally (i.n.) challenged with 50 µl of MV130 [300 Formazin Turbidity Units (FTU)/ml ~ 10^9^ bacteria/ml] or excipient (5% glycerol in PBS) three times a week for 2 weeks as illustrated in **Figure 1A**. Weight and general wellness were monitored before and after each excipient or MV130 administration.

### 2.3 SARS-CoV-2 Coronavirus Infection

On day 2 after the last excipient or MV130 inoculation, K18-hACE2 mice were transferred from the CNIC animal house to the CISA BSL-3 facilities. One week after the last excipient or MV130 inoculation, mice were i.n. infected with 10^4^ PFU of SARS-CoV-2 strain MAD6 (5), kindly provided by José M. Honrubia and Dr. Luis Enjuanes, CNB-CSIC, Madrid, Spain. Mice were ﻿monitored daily for weight, general health, and survival, following the institutional guidance.

### 2.4 Vaccinations

#### 2.4.1 Intramuscular MVA-S

Three weeks after the last excipient or MV130 administration, C57BL/6 mice received an intramuscular inoculation of 10^7^ PFU of sucrose-purified MVA-S virus in 100 µl of PBS (50 µl/leg) as previously described (5). Mice were bled, euthanized, processed, and analyzed after 10 days.

#### 2.4.2 Intranasal MVA-S

Three weeks after the last excipient or MV130 administration, C57BL/6 mice received an i.n. inoculation of 10^7^ PFU of sucrose-purified MVA-S virus in 50 µl of PBS (50 µl/leg). Mice were bled, euthanized, processed, and analyzed 10 days afterwards.

#### 2.4.3 Intranasal S-R848

Three weeks after the last excipient or MV130 administration, C57BL/6 mice received an i.n. inoculation of 10 µg of purified full-length S protein together with 20 µg of R-848 in 50 µl PBS. Alternatively, the same vaccine was also administered 1 week after the last excipient or MV130 administration in a prime-boost scheme. Mice were bled, euthanized, processed, and analyzed 10 days afterwards.

### 2.5 Bronchoalveolar Lavages and Effector Phenotype Analysis

Bronchoalveolar lavage (BAL) were performed in euthanized mice using blunt fill needles (18G; BD) in 700 µl of RPMI 1640 culture medium (Gibco). Collected volumes were centrifuged (1,600 rpm, 4°C, 5 min). Supernatants were kept for mucosal IgA analysis. Cellular pellets were resuspended in FACS buffer for flow cytometry analysis. Immune (CD45^+^) cellular populations were defined as follows: alveolar macrophages (AMs), CD11c^bright^ SIGLEC-F^+^; neutrophils, CD11b^+^ Ly6C^+^ SSC/FSC^high^; cDC1s, CD11c^+^ MHCII^+^ CD103^+^ CD11b^−^; cDC2s, CD11c^+^ MHCII^+^ CD11b^+^ CD103^−^; B cells, B220^+^ MHCII^+^ CD11c^−^ CD90^−^; CD4^+^ T cells, CD90^+^ B220^−^ CD4^+^ CD8^−^; CD8^+^ T cells, CD90^+^ B220^−^ CD8^+^ CD4^−^; CD8^+^ Trms, CD90^+^ B220^−^ CD8^+^ CD4^−^ CD103^+^ CD69^+^.

The effector phenotype in lung and spleen cellular suspensions was determined by gating on CD4^+^ and CD8^+^ T cells as indicated, and analyzing the frequency of CD44^+^ cells inside each population.

### 2.6 Multiparametric Representation of FACS Data

t‐Distributed Stochastic Neighbor Embedding (tSNE), which enables unbiased visualization of high-dimensional similarities of cells in a two-dimensional map, was performed with R (v3.6.3). Briefly, alive AMs and CD45^+^ cells were concatenated using FlowJo, and data from four biological replicates of each experimental condition were imported into R. The same number of cells was considered for any of the experimental conditions. tSNE was performed using the Rtsne package using the default parameters, and FlowSOM (9) was used for the clustering analysis. The discontinued line shown in the figures defines the manual merging of clusters obtained with FlowSOM after the metaclustering step with ConsensusClusterPlus.

### 2.7 Intracellular IFNγ Staining

For intracellular IFNγ determination, as published elsewhere (10), mice received a prime-boost scheme of vaccination, independently of the vaccine to be studied. Thus, 1 week after MV130/excipient administration, mice received a first vaccine dose. A second booster dose of the vaccine was administered after a further 2 weeks. Ten days afterwards, mice were processed and analyzed.

Single-cell suspensions from lung and spleen tissue were plated in 96-well U-bottom plates. One-fifth of the left lung lobule and 2 × 10^6^ splenocytes were cultured in a total volume of 200 µl of RPMI 1640 supplemented with 2 mM L-glutamine, 100 U/ml penicillin, 100 µg/ml streptomycin, 50 µM 2-mercaptoethanol, and 10% heat-inactivated fetal bovine serum (FBS) (all from Life Technologies, Carlsbad, CA, USA) at 37°C. Cell suspensions were restimulated for 6 h with a commercial cocktail of peptides covering the immunodominant domains of the SARS-CoV-2 S protein (Peptivator® SARS-CoV-2 Prot_S; Miltenyi Biotech) at a final concentration of 1 µg/ml, following the manufacturer’s instructions. Brefeldin A (5 µg/ml) was added during the last 4 h of culture. Cells were then collected, stained for surface markers, and intracellular IFNγ staining was performed according to Fixation/Permeabilization Solution kit (BD Cytofix/Cytoperm). IFNγ-producing cells were defined as CD44^+^ IFNγ^+^.

### 2.8 Immunoglobulin Quantification

Serum samples were prepared by incubating blood samples in collection tubes without anticoagulant for 15 min at room temperature to allow clotting to occur. Samples were then centrifuged at 15,000 rpm for 15 min, and serum was moved to a fresh collection tube. Specific immunoglobulins were determined by ELISA as previously described (11). Briefly, S protein or RBD were plated in RIA plates (Corning) in carbonate buffer at 1 µg/ml overnight at 4°C. Plates were washed three times with PBS 0.05% Tween-20 and blocked with PBS supplemented with 10% FBS for at least 1 h at RT. Plates were washed once, and serum was plated at 1/50, 1/250, 1/1,250, and 1/6,250 serial dilutions, while BALs were directly plated at 1/1, 1/3, and 1/9 dilutions in PBS supplemented with 10% FCS, in order to visualize dose-response signals. Samples were incubated overnight at 4°C. Plates were then washed three times with PBS 0.05% Tween-20. Plates containing serum samples were incubated with biotinylated anti-mouse total IgG, IgG1, IgG2c (all from BD) at 2 µg/ml for 1 h at RT followed by three washes with PBS 0.05% Tween-20 and incubation with Streptavidin-HRP (BD) at 1 µg/ml for 30 min at RT. Plates containing BAL samples, after washing, were incubated with 2 µg/ml of an anti-mouse IgA-HRP for 1 h at RT (SouthernBiotech). All plates were then washed three times with PBS 0.05% Tween-20 and developed in the presence of TMB substrate and TMB stop solution (Biogen). Absorbance was read at 450 nm. Data shown correspond to 1/250 for serum and 1/1 for BAL, where no saturation was observed, and are given as Optical Density (OD). Each dot represents an individual mouse.

### 2.9 Statistical Analysis

Mice were included in the studies in a blind manner and randomly assigned to receive excipient or MV130 (1:1 simple randomization) or any of the vaccination regimens.

All statistical analyses were performed using Prism software (GraphPad Software). Statistical significance for comparison between two sample groups with a normal distribution (Shapiro–Wilk test for normality) was determined by unpaired two-tailed Student’s t-test, unless indicated otherwise. In case not following a Gaussian distribution, statistical significance was established by non-parametric Mann–Whitney test. For comparison of weight loss evolution, two-way ANOVA tests were performed. Comparison of survival curves was carried out by Log-rank (Mantel–Cox) test. Outliers were identified by means of Tukey’s range test.

Differences between excipient and MV130 treated mice were considered significant at p < 0.05 (*p < 0.05; **p < 0.01; ***p < 0.001). Differences between vaccinated and non-vaccinated mice under excipient conditions were considered significant at p < 0.05 (#). No * or # indicates non-significant differences.

In figure legends, “N” represents the number of independent experiments performed and “n” the total number of individual mice included in the experiment shown.

## 3 Results

### 3.1 MV130 Protects Against SARS-CoV-2 Experimental Infection

Previous clinical results on MV130 protection against respiratory infections of viral etiology (7) prompted us to investigate whether MV130 could protect against SARS-CoV-2 infection. As a prophylactic mouse model of mucosal MV130 inoculation, C57BL/6 mice were i.n. administered with MV130 or its excipient three times a week for 2 weeks, and rested for 1 week before any further treatment (**Figure 1A**). This administration schedule did not affect the weight (**Supplementary Figure S1A**) or the cellular composition of the blood between the excipient and the MV130-treated groups, except for a slight increase in circulating monocytes in MV130-treated mice (**Supplementary Figure S1B**). Furthermore, no signs of immunopathology were observed in hematoxylin-eosin-stained lung sections after this i.n. administration regimen (**Supplementary Figure S1C**). Of note, the analysis of bronchoalveolar lavages (BAL) showed that MV130 treatment induced an immune (CD45^+^) infiltration in the airways (**Figure 1B**), which included both myeloid (**Figure 1C**) and lymphoid cells (**Figure 1D**), indicating the generation of a pro-inflammatory airway microenvironment.

Next, we evaluated whether MV130 conferred protection against SARS-CoV-2 infection. Thus, K18-hACE2 mice, susceptible to SARS-CoV-2 infection (12, 13), were treated with excipient or MV130 as indicated (**Figure 1A**), and, after 1 week of resting, further challenged (i.n.) with SARS-CoV-2 [MAD6 isolate, 10^4^ plaque-forming units (PFU)/mouse)]. Mice receiving both excipient and MV130 showed slight weight loss, with a trend towards a more limited reduction and better recovery of MV130-treated mice (**Figure 1E**). Notably, at the dose of SARS-CoV-2 used, mortality in the control group (excipient) was 60%, while in the MV130-treated group was reduced to 25% (**Figure 1F**). Therefore, the preventive mucosal administration of MV130 confers protection against SARS-CoV-2 infection.

### 3.2 MV130 Increases the Immunogenicity of an MVA-Based COVID-19 Vaccine Candidate

**Figure 2:**
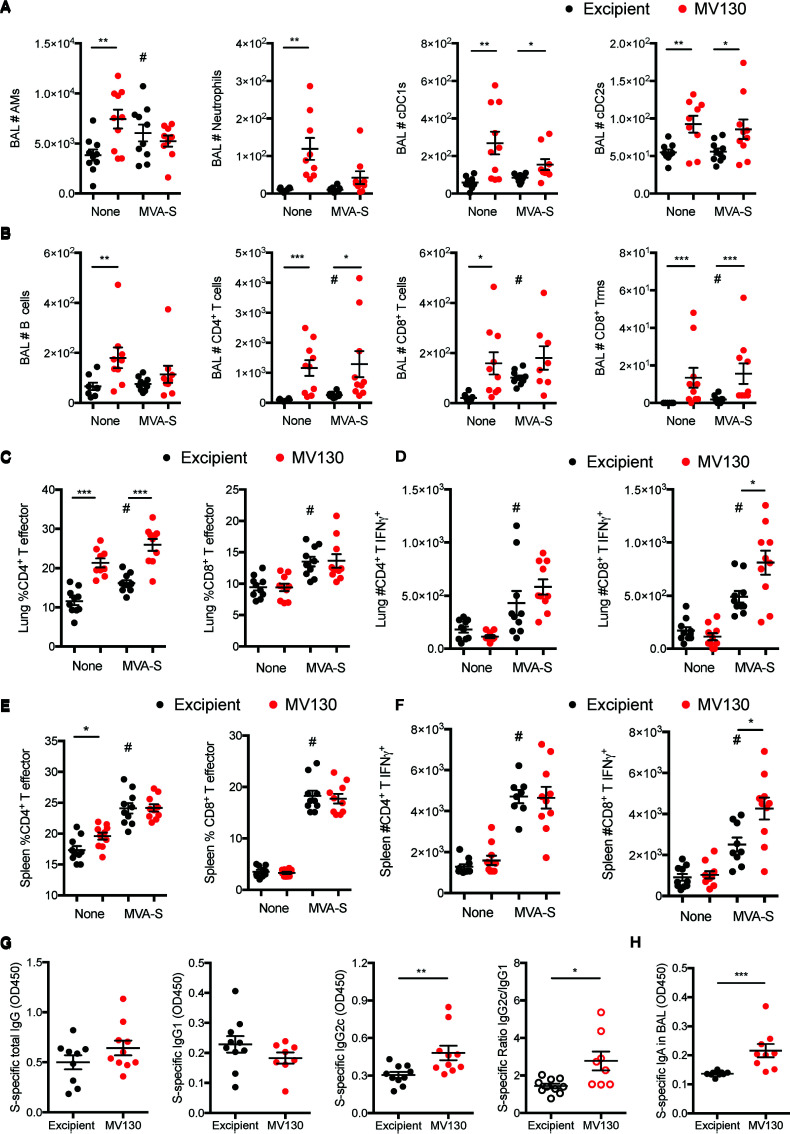
MV130 increases immunogenicity of an MVA-based COVID-19 vaccine candidate. Three weeks after receiving excipient or MV130 according to **Figure 1A**, C57BL/6 mice were i.m. vaccinated with a COVID-19 vaccine based on a Modified Vaccinia virus Ankara (MVA) vector expressing the entire SARS-CoV-2 Spike (S) protein. Samples were collected 10 days later. **(A, B)** Bronchoalveolar lavage (BAL) was obtained from excipient and MV130-treated mice. Quantitative analysis of different myeloid **(A)** or lymphoid **(B)** immune populations identified by FACS. AMs, alveolar macrophages; cDC1s, type 1 conventional dendritic cells; cDC2s, type 2 conventional dendritic cells. **(C, D)** Lungs were recovered, and the effector phenotype (CD44^+^) of CD4^+^ and CD8^+^ T cells was analyzed **(C)**. SARS-CoV-2 S protein-specific activation of CD4^+^ and CD8^+^ T cells was assessed by analyzing intracellular IFNγ expression by FACS **(D)** as described in *Methods*. **(E, F)** The effector phenotype **(E)** and SARS-CoV-2 S protein-specific activation **(F)** of T cells from the spleen were analyzed, as in **(C, D)**. **(G)** Levels of SARS-CoV-2 S protein-specific total IgG, IgG1, IgG2c, and the ratio of IgG2c/IgG1 were analyzed in serum of vaccinated mice. Data correspond to values from sera diluted at 1/250. **(H)** Levels of SARS-CoV-2 S protein-specific mucosal IgA were analyzed in the BAL of vaccinated mice. Data correspond to values from BAL diluted at 1/1. **(A–H)** Data shown as mean ± SEM. N=2. *p < 0.05; **p < 0.01; ***p < 0.001, Student’s t test comparing excipient *versus* MV130. **(A–F)** ^#^p < 0.05 comparing non-vaccinated *versus* MVA-S vaccinated conditions. **(G, H)** OD, optical density.

Previous results from a clinical trial with MV130 showed increased *in vitro* lymphoproliferative responses to influenza antigens, leading us to speculate that MV130 could enhance immunogenicity in response to a previous vaccination or infection by this virus (8). We thus decided to explore whether MV130 could improve SARS-CoV-2 antigen-specific immune responses generated by a COVID-19 vaccine candidate. The MVA-S vaccine is based on the MVA poxvirus vector expressing the entire SARS-CoV-2 S protein, and it has been shown to prevent mortality in susceptible mice infected with SARS-CoV-2 after one or two doses, with one dose being less effective than the two-dose prime-boost vaccination scheme (5). Therefore, we wondered whether a prior administration of MV130 could further improve the protection observed after a single dose of the MVA-S vaccine. Three weeks after the last excipient or MV130 administration (see **Figure 1A** schedule), C57BL/6 mice were i.m. vaccinated with 10^7^ PFU of MVA-S or left unvaccinated. Ten days after vaccination, the cellular analysis of the BAL indicated that the enhanced myeloid (**Figure 2A**) and lymphoid (**Figure 2B**) infiltration induced by MV130 in non-vaccinated mice remained for more than 2 weeks after i.n. administration, suggesting that MV130 exerts a long-lasting pro-immunogenic effect. Conversely, the effect of i.m. MVA-S vaccination on the BAL myeloid (**Figure 2A**) and lymphoid (**Figure 2B**) compartment was modest. Of note, MV130 treatment was the main contributor to the presence of CD4^+^ T effector cells in the lung, whereas CD8^+^ T effector cell lung infiltration was dependent on MVA-S immunization (**Figure 2C**). *In vitro* stimulation of whole lung cell suspensions with a cocktail of peptides containing the immunodominant domains of the SARS-CoV-2 S protein showed that MVA-S vaccination resulted in S antigen-specific IFNγ production by both CD4^+^ and CD8^+^ T cells. Notably, this S-specific response was boosted in CD8^+^ T cells of mice pretreated with MV130 (**Figure 2D**). Systemic analysis of the adaptive response in the spleen showed that pretreatment with MV130 did not affect the CD4^+^ or CD8^+^ T effector phenotype triggered by MVA-S vaccination (**Figure 2E**), but enhanced SARS-CoV-2 S-specific responses in CD8^+^ T cells (**Figure 2F**). Altogether, these data indicate that pretreatment with MV130 (i.n.) improves SARS-CoV-2 antigen-specific cytotoxic responses elicited by single-dose i.m. MVA-S vaccination in mice, both at the local level in the lung and systemically.

An extra layer of protection conferred by the i.m. vaccination with MVA-S is the generation of a potent systemic humoral response (5). The analysis of S-specific antibody titers in the serum of excipient and MV130-treated mice after MVA-S vaccination showed comparable total IgG and IgG1 but higher IgG2c levels, leading to a higher S-specific IgG2c/IgG1 ratio (**Figure 2G**), suggestive of a Th1-like protective humoral response (14). Of note, S-specific IgA antibody titers in BAL were higher in MV130-pretreated mice (**Figure 2H**). Remarkably, the global expression pattern of increased mucosal IgA and systemic IgG2c and IgG2c/IgG1 ratio in mice pretreated with MV130 was also reproduced for SARS-CoV-2 S protein Receptor Binding Domain (RBD)-specific antibodies (**Supplementary Figure S2**). In summary, these data indicate that pretreatment with MV130 (i.n.) enhances specific humoral responses of certain antibody isotypes elicited by single-dose i.m. MVA-S vaccination.

### 3.3 MV130 Improves Immunogenicity of Mucosal COVID-19 Vaccine Candidates

Considering that the i.n. route is part of the natural SARS-CoV-2 infection and given that i.n. MV130 administration contributes to the generation of lung mucosal SARS-CoV-2-specific IgA antibodies and CD8^+^ T cells, we wondered whether MV130 could also improve immunogenicity of vaccine candidates when administered through the i.n. route.

**Figure 3:**
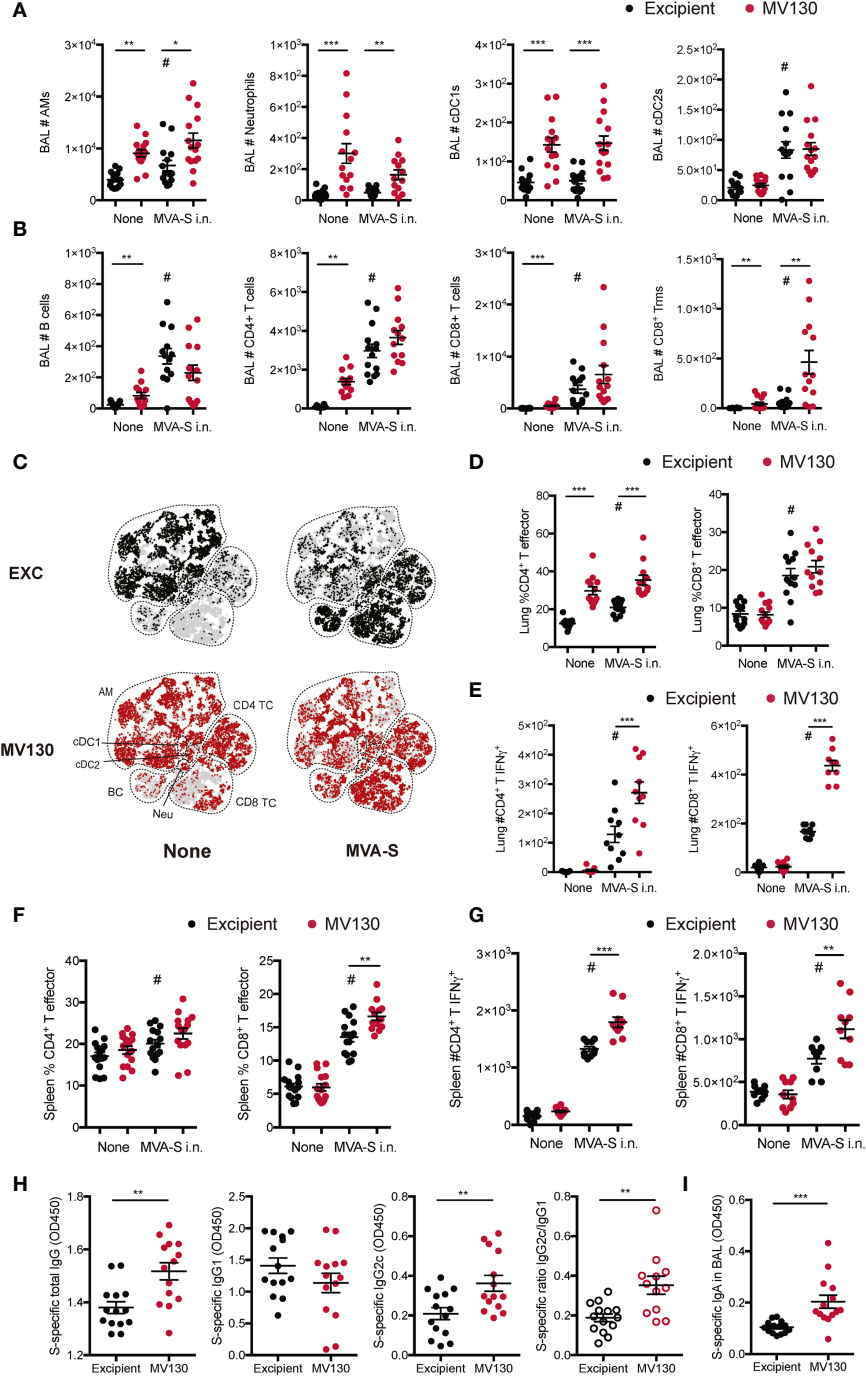
MV130 improves immunogenicity triggered by an MVA-based COVID-19 vaccine candidate after mucosal administration. Three weeks after receiving excipient or MV130 according to **Figure 1A**, C57BL/6 mice were i.n. vaccinated with a COVID-19 vaccine based on a Modified Vaccinia virus Ankara (MVA) vector expressing the entire SARS-CoV-2 Spike (S) protein. Samples were collected 10 days later. **(A, B)** Bronchoalveolar lavage (BAL) was obtained from excipient and MV130-treated mice. Quantitative analysis of different myeloid **(A)** or lymphoid **(B)** immune populations identified by FACS. AMs, alveolar macrophages; cDC1s, type 1 conventional dendritic cells; cDC2s, type 2 conventional dendritic cells. **(C)** BAL cellular composition was analyzed by t-distributed Stochastic Neighbor Embedding (tSNE) analysis from FACS data. Dotted lines identify specific populations. AM, alveolar macrophages; cDC, conventional Dendritic cells; BC, B cells; Neu, neutrophils; TC, T cell. **(D, E)** Lungs were recovered, and the effector phenotype (CD44^+^) of CD4^+^ and CD8^+^ T cells was analyzed **(D)**. SARS-CoV-2 S protein-specific activation of CD4^+^ and CD8^+^ T cells was assessed by analyzing intracellular IFNγ expression by FACS **(E)** as described in *Methods*. **(F, G)** The effector phenotype **(F)** and SARS-CoV-2 S protein-specific activation **(G)** of T cells from the spleen were analyzed, as in **(D, E)**. **(H)** Levels of SARS-CoV-2 S protein-specific total IgG, IgG1, IgG2c, and the ratio of IgG2c/IgG1 were analyzed in serum of vaccinated mice. Data correspond to values from sera diluted at 1/250. **(I)** Levels of SARS-CoV-2 S protein-specific mucosal IgA were analyzed in the BAL of vaccinated mice. Data correspond to values from BAL diluted at 1/1. **(A, B, D–I)** Data shown as mean ± SEM. **(A, B, D, H, I)** N=3. **(E, G)** N=2. **(A, B, D–I)** *p < 0.05; **p < 0.01; ***p < 0.001, Student’s t test comparing excipient *versus* MV130. **(A, B, D–I)** ^#^p < 0.05 statistical significance comparing non-vaccinated *versus* vaccinated conditions. **(H, I)** OD, optical density.

Thus, MV130/excipient-treated C57BL/6 mice were i.n. vaccinated (or not) with 10^7^ PFU of MVA-S 3 weeks after the last dose of MV130/excipient. Analysis of BAL cellular composition 10 days after MVA-S i.n. vaccination revealed increased numbers of alveolar macrophages (AMs) and type 2 conventional dendritic cells (cDC2s) (**Figure 3A**), as well as increased lymphoid infiltration (**Figure 3B**). Consistently, MV130 pretreatment induced a marked infiltration of myeloid cells (**Figure 3A**). Of note, while MV130 only induced a mild lymphoid infiltration in BAL as compared to MVA-S i.n., a clear synergistic effect was seen in CD8^+^ Trm cell induction (**Figure 3B**). t-distributed Stochastic Neighbor Embedding (tSNE) analysis from the BAL confirmed that MVA-S i.n. vaccination increased the relative frequency of the lymphoid compartment in excipient pretreated control mice (**Figure 3C**, upper panels). Interestingly, the most relevant qualitative effect of MV130 administration was the increased proportion of both CD4^+^ and CD8^+^ T cells (**Figure 3C**, lower panel).

The i.n. MVA-S vaccine also induced lung CD4^+^ and CD8^+^ T effector cells, with MV130 pretreatment affecting infiltration of CD4^+^ T effector cells (**Figure 3D**). Remarkably, MV130 immunotherapy boosted the SARS-CoV-2 S-specific activation of both CD4^+^ and CD8^+^ T cells (**Figure 3E**). Similar induction of effector CD4^+^ and CD8^+^ T cells was found in the spleen, with a significant enhancement in CD8^+^ T cells in the presence of MV130 (**Figure 3F**). Of note, similar to the lung tissue, MV130 pretreatment increased the number of splenic S-specific CD4^+^ and CD8^+^ T cells secreting IFNγ after MVA-S i.n. vaccination (**Figure 3G**).

The analysis of humoral responses in mice vaccinated i.n. with MVA-S revealed that MV130 pretreatment increased serum SARS-CoV-2 S protein-specific total IgG and IgG2c antibody titers, resulting in a higher pro-Th1 IgG2c/IgG1 ratio (**Figure 3H**). Notably, MVA-S-vaccinated (i.n.) mice pretreated with MV130 showed a clear enhancement in SARS-CoV-2 S-specific mucosal IgA production in the airways (**Figure 3I**). These responses were similarly conditioned by MV130 when analyzing the production of antibodies against the SARS-CoV-2 S protein RBD (**Supplementary Figure S3**). To sum up, pretreatment with MV130 improves the specific cellular and humoral responses elicited by the MVA-S vaccine candidate when administered i.n.

Next, we wondered whether the capacity of MV130 to boost vaccine immunogenicity could be extended to alternative COVID-19 vaccine formulations. Thus, we formulated an experimental preparation with the soluble full-length SARS-CoV-2 S protein in combination with R848, a Toll-Like Receptor (TLR)-7/8 ligand commonly used as an experimental adjuvant, that mimics the ssRNA of the virus genome (15, 16). The following experiments were performed using S-R848 i.n. with or without a pretreatment with MV130/excipient (i.n.) as depicted in **Figure 1A**.

**Figure 4:**
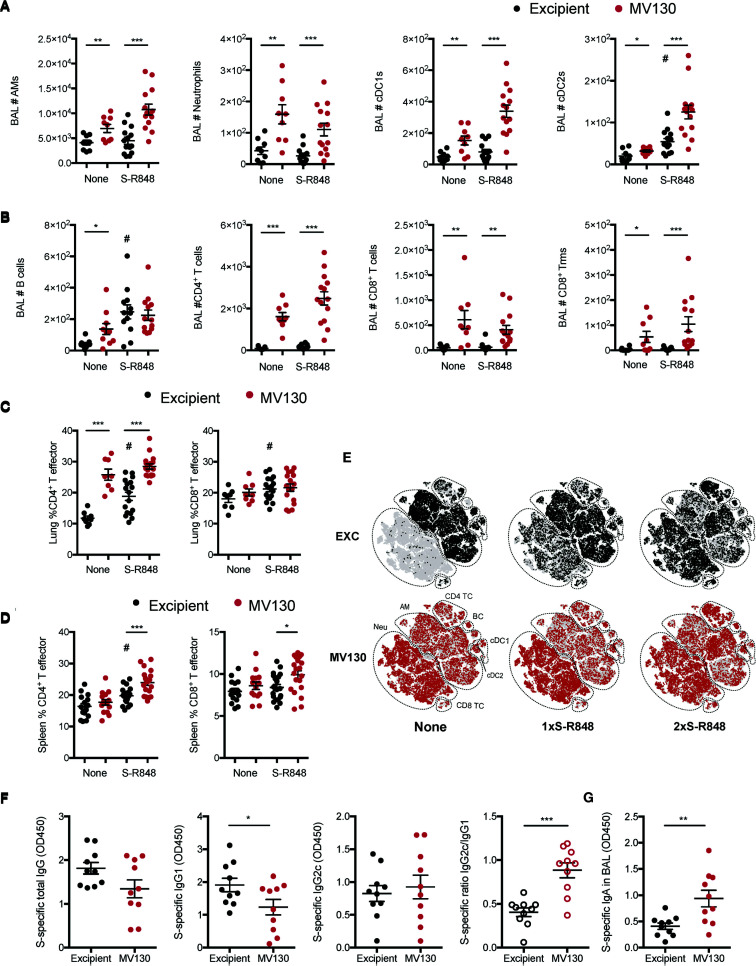
MV130 increases immunogenicity elicited by a mucosal COVID-19 vaccine based on S-protein plus adjuvant. Three weeks after receiving excipient or MV130 according to **Figure 1A**, C57BL/6 mice were i.n. vaccinated with a COVID-19 vaccine based on the entire SARS-CoV-2 Spike (S) protein adjuvanted with R-848. Samples were collected 10 days later. **(A, B)** Bronchoalveolar lavage (BAL) was obtained from excipient and MV130-treated mice. Quantitative analysis of different myeloid **(A)** or lymphoid **(B)** immune populations identified by FACS. AMs, alveolar macrophages; cDC1s, type 1 conventional dendritic cells; cDC2s, type 2 conventional dendritic cells. **(C, D)** Lungs **(C)** and spleen **(D)** were recovered, and the effector phenotype (CD44^+^) of CD4^+^ and CD8^+^ T cells were analyzed. **(E–G)** Excipient and MV130-treated mice were i.n. prime-vaccinated with S-R848 1 week after the last immunotherapy inoculation. Two weeks later, mice were administered with a second booster dose of the vaccine. **(E)** BAL cellular composition was analyzed by t-distributed Stochastic Neighbor Embedding (tSNE) analysis from FACS data. This analysis included non-vaccinated mice together with mice vaccinated with one (1×) or two (2×) doses of the S-R848 vaccine. Dotted lines identify specific populations. AM, alveolar macrophages; cDC, conventional Dendritic cells; BC, B cells; Neu, neutrophils; TC, T cell. **(F)** Levels of SARS-CoV-2 S protein-specific total IgG, IgG1, IgG2c, and the ratio of IgG2c/IgG1 were analyzed in serum of vaccinated mice. Data correspond to values from sera diluted at 1/250. **(G)** Levels of SARS-CoV-2 S protein-specific mucosal IgA were analyzed in the BAL of vaccinated mice. Data correspond to values from BAL diluted at 1/1. **(A–D, F–G)** Data shown as mean ± SEM. **(A–D)** N=3. **(F, G)** N=2. **(A–D, F, G)** *p < 0.05; **p < 0.01; ***p < 0.001, Student’s t test comparing excipient *versus* MV130. **(A–D)** ^#^p < 0.05 Student’s t test comparing non-vaccinated *versus* vaccinated conditions. **(F, G)** OD, optical density.

BAL analysis 10 days after vaccination showed that i.n. S-R848 vaccination had a mild effect on cell infiltration, increasing the numbers of only cDC2 and B cells (**Figures 4A, B**). Notably, S-R848 vaccinated mice that had received MV130 immunotherapy had increased numbers of AMs, cDC1s, cDC2s, and CD4 T cells (**Figures 4A, B**), suggesting a synergistic pro-immunogenic effect of the vaccine combined with MV130.

S-R848 vaccination induced a milder CD4^+^ T cell effector phenotype than MV130 alone in the lung (**Figure 4C**), while at the systemic level, MV130 pretreatment enhanced both CD4+ and CD8+ effector T cells when combined with the vaccination (**Figure 4D**). The lack of a robust induction of an effector response with S-R848 vaccination was confirmed by the negligible SARS-CoV-2 S protein-specific responses detected both in the lung and spleen (data not shown).

To increase the immunogenicity of S-R848, a prime and boost vaccination scheme was used. One week after the last dose of MV130/excipient treatment, mice were vaccinated (i.n.) with the first dose of S-R848 (following **Figure 1A** schedule). Two weeks later, mice were administered with a second i.n. booster dose of the S-R848 vaccine. The tSNE representation showed that this prime and boost regimen generated notable changes in the frequency of different immune subsets in the BAL, compared with a single dose i.n. S-R848 vaccination (**Figure 4E**, upper panels). MV130 pretreatment enhanced immune cell infiltration in the BAL when given alone or in combination with a single dose of S-R848 or the prime-boost S-R848 vaccination strategy (**Figure 4E**, lower panels). Notably, the evaluation of systemic humoral responses against SARS-CoV-2 S protein under the prime-boost S-R848 vaccination scheme indicated that mice receiving MV130 produced reduced levels of specific IgG1 isotype, resulting in an enhanced pro-Th1 IgG2c/IgG1 ratio (**Figure 4F**). In addition, the levels of SARS-CoV-2 S protein-specific mucosal IgA in the BAL were higher in MV130 pretreated mice (**Figure 4G**). In summary, these data suggest that pretreatment with MV130 increases specific immune responses to SARS-CoV-2 antigens triggered by different COVID-19 vaccine candidates administered *via* the i.n. route.

## 4 Discussion

Prophylactic strategies directed to exploit the capacity of the innate immune system to be “trained” against viral infections in a heterologous manner could precede the development of antigen-specific vaccines and enhance their immunogenicity upon the expansion of new emerging viruses (17). The tuberculosis vaccine Bacillus Calmette-Guérin (BCG) has been proposed as an inducer of heterologous protective responses against the SARS-CoV-2 coronavirus infection (18). Indeed, a retrospective study suggests that BCG vaccination may be associated with a decrease in COVID-19 incidence (19). Similarly, mucosal administration of the polybacterial preparation MV130 is protective against viral bronchiolitis in children (7). Herein, we demonstrate that prophylactic treatment with MV130 confers heterologous protection against SARS-CoV-2 infection as has been suggested for BCG. Respiratory delivery of BCG was more effective at training innate immune cells compared to intradermal delivery (20), suggesting the potential benefit of this mucosal administration route to generate heterologous protection. Of note, we find that the i.n. administration of MV130 generates a pro-immunogenic cellular microenvironment in the airways, in line with its potential role in activation of antigen-presenting cells and adaptive immunity (21). These results concur with previous studies showing that “non-specific” mucosal immunotherapies show relevant efficacies against respiratory viral infections. For instance, i.n. administration of the immunomodulator Hiltonol, a TLR3 ligand, protected mice from lethal SARS-CoV infection when given both in prophylactic and therapeutic regimens (22). Moreover, considering that acute SARS-CoV-2 infection is linked to leukopenia both in patients (23, 24) and in the K18-hACE2 mouse model used in our study (13), MV130 could be generating and/or maintaining a beneficial pro-immunogenic airway environment, ready to respond to SARS-CoV-2, early upon infection.

Our data support the notion that MV130 improves the immunogenicity of vaccines, as hypothesized in a previous clinical trial (8). MV130 pretreatment boosted the induction of an immunogenic environment in the airways, superior effector T cells and antigen-specific responses, higher antigen-specific pro-Th1 IgG2c/IgG1 ratio, and increased antigen-specific mucosal IgA. The high IgG2c/IgG1 ratio has been linked to protective Th1-related responses, of particular relevance during parasitic (25) and viral (14) infections. Furthermore, this high IgG2c/IgG1 profile is associated with improved cytotoxic responses triggered by CD8^+^ T cells (26, 27). Consistently, prophylactic mucosal MV130 increases SARS-CoV-2-specific IFNγ CD8^+^ T cells elicited by MVA-S vaccination, not only in the lungs but also in the spleen. This effect is likely boosted by the enhanced CD4^+^ effector T cell phenotype (6). The high pro-Th1 skewing induced by MV130 may also be crucial to prevent maladaptive responses, described predominantly in aged individuals after vaccination against SARS-CoV-2 (28). Therefore, prophylaxis with MV130 generates a concerted pro-immunogenic environment that may improve adaptive immune responses triggered by COVID-19 vaccine candidates. This enhanced response deserves further exploration in clinical studies to address the potential benefits of prophylactic regimens of MV130 administration for responses to a given vaccine, particularly in the elderly population (29).

Our results concur with former observations regarding the capacity of MV130 to promote Th1 responses in mixed lymphocyte reactions between human monocyte-derived dendritic cells and naïve CD4^+^ T cells (21). The signaling adaptors Receptor-Interacting serine/threonine-Protein Kinase-2 (RIPK2) and Myeloid Differentiation primary response 88 (MyD88) were mechanistically implicated in this pro-inflammatory effect (21). Note that it is well established that the recognition of microbial-associated molecular patterns by the receptors Nucleotide-binding Oligomerization Domain-containing protein-1, -2 (NOD1, NOD2), and TLRs (*via* MyD88) leads to RIPK2 activation (30). Interestingly, BCG induces innate immune memory through RIPK2 (31). Therefore, sensing of MV130 by these receptors and the triggering of RIPK2-mediated signaling pathways may be involved in the induction of trained immunity by MV130. In addition, the study of RIPK2 as a potential common mediator of innate immune memory merits further study.

Our results also indicate that SARS-CoV-2-specific mucosal IgA titers in the airways after vaccination are significantly increased in the presence of MV130. Of note, this was observed in all three types of vaccination tested, regardless of the route used for administration of the vaccine or its intrinsic immunogenicity. This points to the potential of MV130 prophylaxis to reinforce mucosal immunity, which is critical for the protection against respiratory viral infections, including coronaviruses. For instance, the intranasal vaccination against SARS-CoV limited viral replication in the lungs, correlating with the titers of SARS-CoV-specific IgA (32). This is also the case for protection against SARS-CoV-2 in hamsters (33) and mice (10). In addition, it is becoming clear that mucosal IgA both in saliva (34) and nasopharynx (35) is fundamental to neutralize SARS-CoV-2 infection and prevent its spread.

In conclusion, our results indicate that mucosal immunotherapy with MV130 represents a promising new tool to fight the tremendous global challenge represented by new emerging viruses, exemplified by SARS-CoV-2. Its prophylactic administration shows a double benefit, both inducing *per se* heterologous protection against SARS-CoV-2 infection and boosting the immunogenicity of antigen-specific COVID-19 vaccine candidates. This broad beneficial effect of MV130 administration likely relies on the capacity of this immunotherapy of targeting both innate and adaptive immune responses, improving vaccines with different immunogenic capacity (**Supplementary Figure S4**), meeting the criteria of a trained-immunity-based vaccine (17). Moreover, these heterologous prophylactic interventions, given the lack of antigen dependence, hold promise for potential early administration in future outbreaks of emerging viruses.

## Data Availability Statement

The raw data supporting the conclusions of this article will be made available by the authors, without undue reservation.

## Ethics Statement

The animal study was reviewed and approved by Centro Nacional de Investigaciones Cardiovasculares (CNIC), Madrid, Spain; Centro de Investigación en Sanidad Animal (CISA), Madrid, Spain; Division of Animal Protection of the Comunidad de Madrid (PROEX 169.4/20, PROEX 237-16, PROEX 115/19 and PROEX240-16). Animal procedures were conformed to Spanish law under the Royal Decree (RD 53/2013) and in accordance to EU Directive 2010/63EU and Recommendation 2007/526/EC.

## Author Contributions

CF, JG-A, ME, and DS conceived and designed the laboratory experiments. CF, JG-A, SM-C, AJ-C, PB, JA-I, UG, and PG performed laboratory experiments. IH-M and GD performed essential data analysis. CS-C, MP, NV, AL-P, SC, AG-C, OL, and JA-C generated and provided S protein. CF and DS analyzed and interpreted the laboratory experiments. CF wrote the initial draft that was edited by DS. All authors reviewed and revised the manuscript prior to submission. All authors contributed to the article and approved the submitted version.

## Funding

CF was supported by AECC Foundation (INVES192DELF) and is currently funded by the Miguel Servet program (ID: CP20/00106) (ISCIII). IH-M receives the support of a fellowship from la Caixa Foundation (ID 100010434, fellowship code: LCF/BQ/IN17/11620074) and from the European Union’s Horizon 2020 research and innovation program under the Marie Skłodowska-Curie grant agreement no. 713673. AJ-C is a postgraduate fellow of the City Council of Madrid at the Residencia de Estudiantes (2020–2021). GD is supported by an European Molecular Biology Organization (EMBO) Long-term fellowship (ALTF 379-2019). This project has received funding from the European Union's Horizon 2020 research and innovation programme under the Marie Skłodowska-Curie grant agreement No. Project number 892965. OL and JA-C acknowledge Comunidad de Madrid (Tec4Bio-CM, S2018/NMT-4443, FEDER). Work in OL laboratory was funded by CNIO with the support of the projects Y2018/BIO4747 and P2018/NMT4443 from Comunidad de Madrid and co-funded by the European Social Fund and the European Regional Development Fund. The CNIO is supported by the Instituto de Salud Carlos III (ISCIII). Work at CNB and CISA is funded by the Spanish Health Ministry, Instituto de Salud Carlos III (ISCIII), Fondo COVID-19 grant COV20/00151, and Fondo Supera COVID-19 (Crue Universidades-Banco Santander) (to JG-A). Work in the DS laboratory is funded by the CNIC; by the European Research Council (ERC-2016-Consolidator Grant 725091); by Agencia Estatal de Investigación (PID2019-108157RB); by Comunidad de Madrid (B2017/BMD-3733 Immunothercan-CM); by Fondo Solidario Juntos (Banco Santander); by a research agreement with Inmunotek S.L.; and by Fundació La Marató de TV3 (201723). The CNIC is supported by the Instituto de Salud Carlos III (ISCIII), the MICINN, and the Pro CNIC Foundation.

## Conflict of Interest

SM-C is an employee of Inmunotek S.L. DS laboratory holds a collaboration agreement between CNIC and Inmunotek.

The remaining authors declare that the research was conducted in the absence of any commercial or financial relationships that could be construed as a potential conflict of interest.

## Publisher’s Note

All claims expressed in this article are solely those of the authors and do not necessarily represent those of their affiliated organizations, or those of the publisher, the editors and the reviewers. Any product that may be evaluated in this article, or claim that may be made by its manufacturer, is not guaranteed or endorsed by the publisher.
