# Supplementary material for: The Bacterial Mucosal Immunotherapy MV130 Protects Against SARS-CoV-2 Infection and Improves COVID-19 Vaccines Immunogenicity
Source: Front Immunol. 2021 Nov 18;12:748103. doi: 10.3389/fimmu.2021.748103 (PMC8637175; doi:10.3389/fimmu.2021.748103)
Supplement: Supplementary file 1 [file DataSheet_1.pdf]

Supplementary Material

1 Supplementary figures

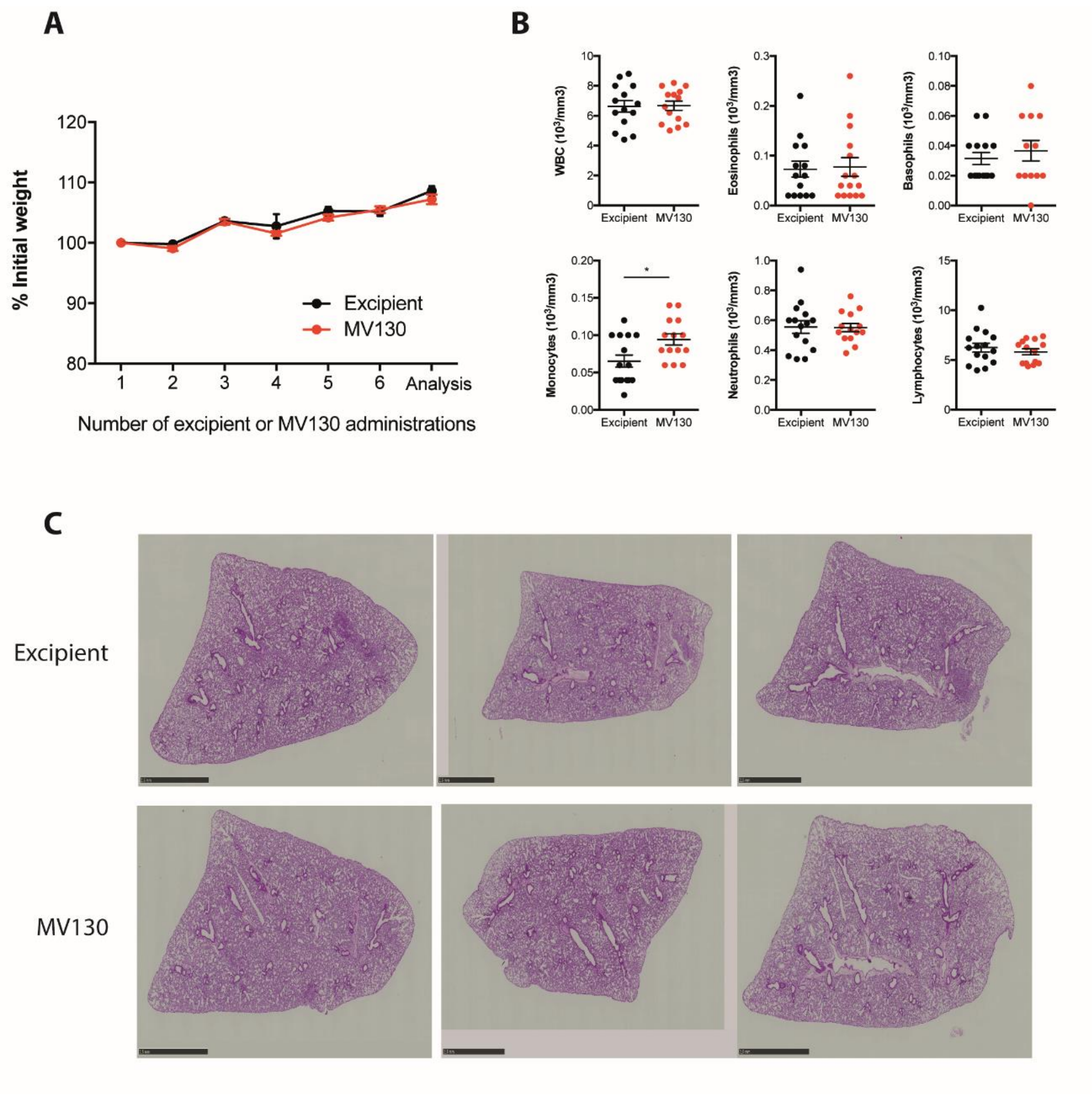

Supplementary Figure S1. MV130 administration does not show adverse effects on mouse wellbeing, related to Figure 1. Mice were intranasally administered with excipient or MV130 three times per week for two weeks according to Figure 1A. **(A)** Weight was monitored during this treatment until further analysis. **(B)** One week after the last excipient or MV130 administration, mice were bled and blood counts were analyzed. WBC: White Blood Cells. (A,B) Data shown as mean ± SEM. N=2, (A) n=20, (B) n=15. *$p<0.05$ Student's t test comparing excipient versus MV130. **(C)** Lungs were collected, paraffin-embedded and sections were stained with hematoxylin-eosin to visualize signs of immunopathology. Representative images of 3 mice per experimental condition are shown. Scale bar = 2.5 mm.

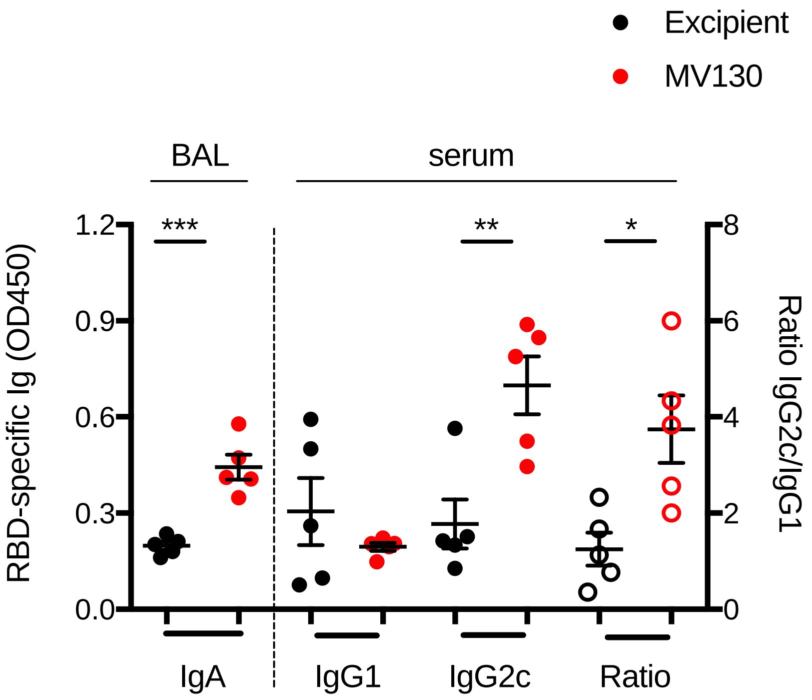

Supplementary Figure S2. MV130 improves humoral immunity against the SARS-CoV-2 S protein RBD elicited by an MVA-based intramuscular COVID-19 vaccine candidate, related to Figure 2. Three weeks after receiving excipient or MV130 according to Figure 1A, C57BL/6 mice were vaccinated intramuscularly with a COVID-19 vaccine based on a Modified Vaccinia virus Ankara (MVA) vector expressing the entire SARS-CoV-2 Spike (S) protein. Ten days afterwards, bronchoalveolar lavage (BAL) and serum were collected and specific antibodies against the Receptor Binding Domain (RBD) of the SARS-CoV-2 S protein were determined by ELISA. Mucosal IgA was detected in BAL (left). Levels of IgG1, IgG2c (middle) and the ratio between IgG2c/IgG1 (right) were determined in serum. Data correspond to values from sera diluted at 1/250 and from BAL diluted at 1/1. Data shown as mean ± SEM. *p<0.05; **p<0.01; ***p< 0.001, Student's t test comparing excipient versus MV130. OD: optical density.

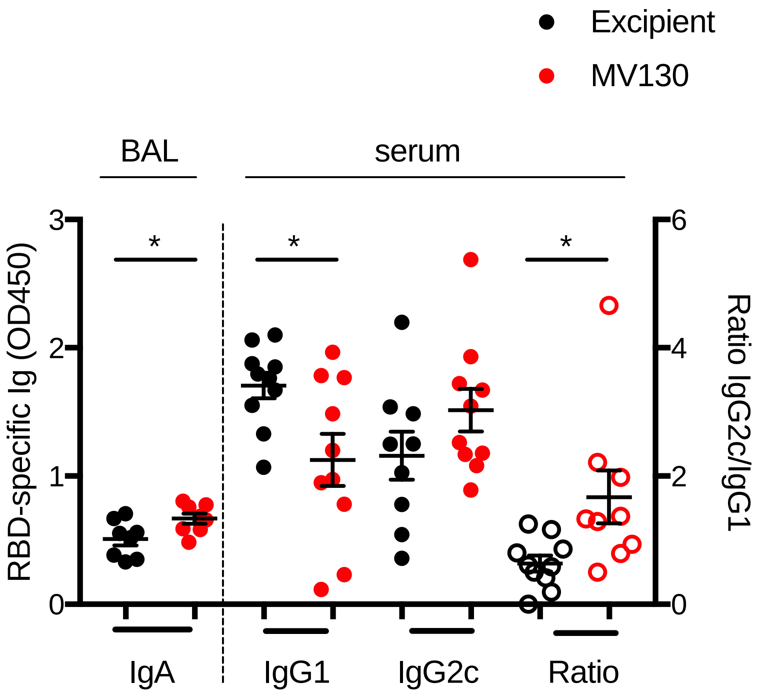

Supplementary Figure S3. MV130 improves humoral immunity against the SARS-CoV-2 S protein RBD elicited by an MVA-based COVID-19 vaccine candidate administered intranasally, related to Figure 3. Three weeks after receiving excipient or MV130 according to Figure 1A, C57BL/6 mice were intranasally vaccinated with a COVID-19 vaccine based on a Modified Vaccinia virus Ankara (MVA) vector expressing the entire SARS-CoV-2 Spike (S) protein. Ten days afterwards, bronchoalveolar lavage (BAL) and serum were collected and specific antibodies against the Receptor Binding Domain (RBD) of the SARS-CoV-2 S protein were determined by ELISA. Mucosal IgA was detected in BAL (left). Levels of IgG1, IgG2c (middle) and the ratio between IgG2c/IgG1 (right) were determined in serum. Data correspond to values from sera diluted at 1/250 and from BAL diluted at 1/1. Data shown as mean ± SEM. *$p<0.05$; Student's t test comparing excipient versus MV130. OD: optical density.

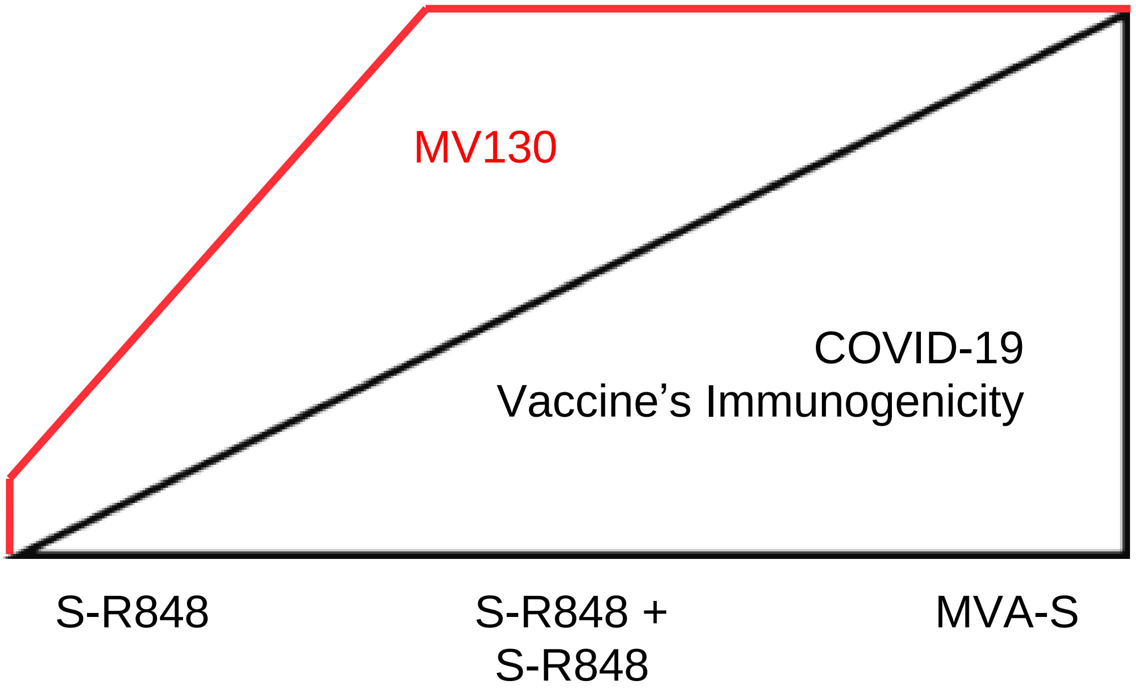

Supplementary Figure S4. Model for the capacity of MV130 to improve the immunogenicity of COVID-19 vaccine candidates. Depending on their formulation, COVID-19 vaccines can trigger different levels of immunogenicity, ranging from mild as shown with a single dose of the S-R848 vaccine candidate, to high such as the MVA-S vaccine candidate. The prophylactic administration of the mucosal immunotherapy MV130 improves immunogenic responses elicited by those vaccine formulations with room for improvement, suggesting that combination with prophylactic interventions could enhance the immunogenicity of suboptimally effective vaccines.

2 Supplementary Materials and methods

2.1 Hematology

One week after the last excipient or MV130 administration, C57BL/6 mice were bled and blood count analysis was performed by the Comparative Medicine Unit at CNIC in a PENTRA 60 hematology analyzer.

2.2 SARS-CoV-2 S and RBD protein expression and purification

Plasmids for expression of SARS-CoV-2 RBD or full-length S protein including mutations to remove the cleavage site (RRAR to AAAA), stabilize the protein (K986P and V987P), and include a C-terminal hexahistidine tag, were kindly provided by Dr. Florian Krammer (Icahn School of Medicine, Mt. Sinai) (1). DNA for transfection was produced using Qiagen Plasmid Giga Kit following manufacturer's instructions.

Expression of proteins was carried out in Expi293F cells (Thermo Fisher Scientific). Briefly, cells grown in Expi293 Expression Medium (Thermo Fisher Scientific) were seeded at 3 x 10^6 cells/ml, and grown at 37ºC and 5% CO_2. After 24 hours, cells were diluted to 3 x 10^6 cells/ml, transfected using Expifectamin 293 Transfection Kit (Thermo Fisher Scientific) or PEI Max (Linear Polyethylenimine Hydrochloride, Polysciences) transfection reagent and grown at 32 ºC and 8% CO_2. 72 hours after transfection, cells were harvested and the supernatant was collected, filtered through 0.45 μm and 0.22 μm PVDF membranes (Jet Biofil), and supplemented with protease inhibitors (cOmplete, EDTA-free Protease Inhibitor Cocktail, Roche).

Protein purification was achieved following guidelines previously described (2,3) with several modifications. 4-6 mL of Ni-NTA agarose slurry (Qiagen) per 100 mL of supernatant were equilibrated in E/W buffer (50 mM NaPi pH 7, 300 mM NaCl) or modified PBS (1.1 mM KH_2PO_4, 3 mM Na_2HPO_4, 155.2 mM NaCl, pH 7.4). Freshly collected culture supernatants were incubated with the equilibrated resin with gentle rocking for either 1 hour at 4ºC (for RBD) or for 3 hours at RT (for spike). Then, the resin was washed with the corresponding equilibration buffer, settled for affinity chromatography, and further washed with equilibration buffer containing 20 mM imidazole. Elution was carried out with appropriate equilibration buffer including either 250 or 500 mM imidazole. Eluted fractions were pooled together and concentrated using Amicon Ultra-0.5 Centrifugal Filter Units 30 K (for RBD) or Amicon Ultra-2 Centrifugal Filter Units 30 K (for spike) (Millipore). The concentrated proteins were then filtered with an UltraFree MC GV Filter (Millipore) prior to size-exclusion chromatography. FPLC purification was performed in an ÄKTA Pure 25L system at 4ºC using Superdex 200 Increase 10/300 GL (preferred for RBD) or Superose 200 Increase 10/300 GL (more suitable for spike) columns (GE Healthcare). Standard PBS (137 mM NaCl, 10 mM Na_2HPO_4-$2H_2O$, 2.7 mM KCl, 1.8 mM KH_2PO_4, pH 7.4) was used as the recovery buffer for RBD, whereas S protein was eluted in modified PBS. FPLC fractions were analyzed by SDS-PAGE and those containing the purified protein were pooled together to make 50-µg-protein aliquots, which were snap frozen in liquid nitrogen and stored at -80ºC. Protein concentration was determined by measuring the absorbance at 280 nm in a NanoDrop ND-1000 Spectrophotometer (Thermo Fisher Scientific) using theoretical extinction coefficients (1.213 $(g/L)^{-1}cm^{-1}$ and 1.013 $(g/L)^{-1}cm^{-1}$ for RBD and S proteins, respectively).

2.3 Flow cytometry Methods

2.3.1 Cell suspension preparation

To obtain cell suspensions for flow cytometry, lungs and spleens were digested with Liberase TL (Roche) for 30 or 10 minutes, respectively, at 37ºC and filtered through 70-µm cell strainers (BD Biosciences). In both cases, red blood cells were lysed using RBC Lysis Buffer (Sigma) for 3 minutes at room temperature (RT). Washes and phenotypic analysis of leukocytes were performed on ice-cold FACS Buffer (PBS supplemented with 5 mM EDTA and 3% FBS). Cells were incubated with LIVE/DEAD Fixable AQUA dead cell stain (Thermo Fischer) in PBS for 30 minutes at RT protected from light. Cells were then washed in ice-cold FACS buffer and incubated with indicated antibodies and purified anti-FcɣRIII/II to block Fc-receptors at 4°C for 10 minutes. All analyses were performed in LIVE/DEAD Fixable AQUA dead cell stain-negative cells.

2.3.2 Absolute numbers quantification

The quantification of absolute numbers in immune infiltrates was performed by acquiring a controlled volume of sample corresponding to a fixed volume of digested tissue at a constant speed for the same duration. This method was validated with the use of quantification beads in some of the experiments. Determination of absolute numbers allowed to pool data from the same experimental conditions obtained in independent experiments. In all experiments showing FACS data, each dot represents a single mouse.

2.4 Histopathology

Left lung lobules were collected, fixed in 4% PFA (Thermo Fisher Scientific, Walthman, MA) for an overnight in gentle agitation and kept in PBS at 4º until processed by the Histopathology unit at CNIC. Samples were dehydrated with ethanol, embedded in paraffin and sections (3-5-µm thickness) were stained with hematoxylin and eosin (H&E, Sigma) following standard protocols. Tissue sections were visualized, and images captured under a Leica DM2500 microscope (Leica Microsystems, Solms, Germany).

3 Supplementary References

1. Amanat F, Stadlbauer D, Strohmeier S, Nguyen THO, Chromikova V, McMahon M, Jiang K, Arunkumar GA, Jurczyszak D, Polanco J, et al. A serological assay to detect SARS-CoV-2 seroconversion in humans. *Nat Med* (2020) **26**:1033–1036. doi:10.1038/s41591-020-0913-5
2. Esposito D, Mehalko J, Drew M, Snead K, Wall V, Taylor T, Frank P, Denson JP, Hong M, Gulten G, et al. Optimizing high-yield production of SARS-CoV-2 soluble spike trimers for serology assays. *Protein Expr Purif* (2020) **174**: doi:10.1016/j.pep.2020.105686
3. Stadlbauer D, Amanat F, Chromikova V, Jiang K, Strohmeier S, Arunkumar GA, Tan J, Bhavsar D, Capuano C, Kirkpatrick E, et al. SARS-CoV-2 Seroconversion in Humans: A Detailed Protocol for a Serological Assay, Antigen Production, and Test Setup. *Curr Protoc Microbiol* (2020) **57**: doi:10.1002/cpmc.100
